# Early-life intervention with *Lactobacillus reuteri* enhances intestinal barrier function and resilience in suckling piglets via modulation of gut microbiota and metabolites

**DOI:** 10.3389/fmicb.2026.1791848

**Published:** 2026-04-10

**Authors:** Qin Luo, Yanping Zhang, Jing Liang, Shuang Liang, Qin Xia, Cong Liu, Ganqiu Lan, Yunxiang Zhao, Yutuo Wei

**Affiliations:** 1 College of Animal Science and Technology, Guangxi University, Nanning, China; 2 Guangxi Key Laboratory of Animal Breeding and Disease Control and Prevention, Nanning, China; 3 College of Life Sciences and Technology, Guangxi University, Nanning, China

**Keywords:** gut microbiota, immune function, intestinal barrier, *Lactobacillus reuteri*, metabolomics, suckling piglets

## Abstract

**Background:**

Early-life nutritional interventions are pivotal for promoting piglet growth and health, particularly in reducing weaning-associated disorders.

**Methods:**

This study investigated the effects of early-life *Lactobacillus reuteri* (*L.reuteri*) supplementation on growth performance, immune function, intestinal morphology, gut microbiota, ileal metabolites, and barrier function in suckling piglets. Neonatal piglets were administered *L.reuteri* for either 3 or 7 days post-birth.

**Results and discussion:**

Results indicated that *L.reuteri* supplementation significantly increased weaning weight and average daily gain (ADG) while markedly reducing diarrhea incidence. Immune and antioxidant capacities were enhanced, evidenced by elevated serum and ileal IgG, IL-4, IL-10, T-SOD, T-AOC, and GSH-Px levels, alongside decreased pro-inflammatory cytokines (IL-1β, IL-6, TNF-α) and MDA. Histological analysis revealed improved intestinal architecture, characterized by increased ileal villus height and reduced crypt depth. 16S rRNA sequencing and metabolomic analyses showed that *L.reuteri* reshaped the gut microbiota by expanding beneficial *Lactobacillus* species and suppressing potential pathogens (*Streptococcus, Pasteurellaceae*), while modulating ileal metabolites involved in amino acid and energy metabolism. Multi-omics integration highlighted coordinated interactions between microbial composition and metabolites linked to improved health outcomes. Furthermore, the expression of tight junction proteins (Occludin, Claudin-3, ZO-1) and mucins (MUC-1, MUC-2) was significantly upregulated, indicating strengthened intestinal barrier integrity. Collectively, these findings demonstrate that early-life intervention with *L.reuteri* confers comprehensive benefits on suckling piglet health through immune enhancement, antioxidant protection, microbiota remodeling, metabolic regulation, and barrier reinforcement, supporting its potential as a practical strategy to improve early-life resilience and mitigate weaning-associated disorders in swine production.

## 1 Introduction

In modern intensive swine production, the neonatal phase represents a critical developmental window (Wu et al., 2018). The early postnatal period is particularly important because rapid growth and gastrointestinal maturation establish the foundation for efficient nutrient utilization and long-term health (Tian et al., 2018; Chen et al., 2021). The intestine serves not only as the primary organ for nutrient digestion and absorption but also as a crucial barrier preventing the translocation of exogenous pathogens (Wu et al., 2018). However, suckling piglets are highly vulnerable during this stage due to immature intestinal structures, unstable microbial colonization, and underdeveloped immune responses. Consequently, they are more susceptible to pathogens such as *Escherichia coli* and *Clostridium spp*., resulting in diarrhea, reduced growth performance, and increased mortality (Yang et al., 2017; Huang et al., 2022; Hu et al., 2024).

Early colonization of the gut microbiota plays a pivotal role in shaping host health by facilitating nutrient absorption (Gao et al., 2020), producing essential metabolites like short-chain fatty acids (SCFAs), and facilitating immune system maturation, thereby strengthening intestinal barrier function (Selma-Royo et al., 2020; Jia et al., 2021b; Dowley et al., 2022; Yang et al., 2022). Establishing a balanced and diverse gut micro biota during early life is crucial for the development of homeostatic processes, including the immune system, digestive system, and metabolic processes (Clarke et al., 2014). Beneficial microbes, such as *Lactobacillus* and *Bifidobacterium*, transmitted via colostrum and maternal secretions, form the first line of defense against pathogens (Pham et al., 2017). Nonetheless, environmental stressors and prophylactic antibiotic exposure in intensive systems can disrupt microbial establishment and increase disease susceptibility (Yu et al., 2018; Bi et al., 2022).

Probiotics have emerged as a promising nutritional strategy to promote early-life gut health. Defined as live microorganisms that confer health benefits when administered in adequate amounts, probiotics are considered safe and effective alternatives to antibiotics (Hill et al., 2014; Liu et al., 2021). Their functions include promoting nutrient absorption, preventing diarrhea, reducing inflammation (Butel, 2014; Jha et al., 2020), producing beneficial metabolites, and supporting intestinal barrier integrity (Jiang et al., 2024). They also regulate the balance of the gut microbiota, reduce pathogenic microorganisms (González-Orozco et al., 2023), thereby reducing the incidence of gastrointestinal diseases such as rotavirus diarrhea, inflammatory bowel diseases, and necrotizing enterocolitis (Vanderpool et al., 2008; Azagra-Boronat et al., 2020; Kalantar et al., 2025).

Common probiotic species encompass *Bifidobacterium, Lactobacillus*, yeasts, *Bacillus*, and *Clostridium butyricum*, among others (Karimi et al., 2018; Yao et al., 2024). Among these, Lactobacillus stands out as the most extensively studied probiotic. Its species, which are natural inhabitants of the porcine gut, play a crucial role in human health by preventing intestinal infections and eliminating pathogens (Xu et al., 2022). Its beneficial mechanisms involve the production of organic acids that lower intestinal pH, and the secretion of various metabolites that inhibit the growth of numerous pathogens, hydrogen peroxide, bacteriocins, and carbon peroxide; the blocking of adhesion sites on the intestinal epithelial surface; competion for nutrients; and the stimulation of immune responses (Dec et al., 2018; Rodrigues et al., 2024).

Among lactic acid bacteria, Lactobacillus reuteri has garnered significant attention due to its multifaceted probiotic properties. These benefits include lowering intestinal pH to inhibit pathogens and competitively excluding harmful bacteria (Du et al., 2023). Notably, *L.reuteri* is distinguished by its production of broad-spectrum antimicrobial compounds such as reuterin, its effective gut colonization, and its provision of multiple functional benefits (Coelho et al., 2022; Yu et al., 2023). Supplementation with *L.reuteri KT260178* enhances colonization, growth, and immune function in piglets (Yang et al., 2020), while maternal *L.reuteri* can further support gut microbial maturation through vertical transmission (Wang et al., 2022). Research has also demonstrated that *L.reuteri* can enhance intestinal barrier function and maintain tight junction protein integrity, regulate gut microbiota, and improve inflammation. Supplementing with *L.reuteri* LR1 has been shown to improve intestinal morphology and barrier function in weaned piglets (Yi et al., 2018). *L.reuteri* XY227 enhances growth performance in fattening pigs and regulates lipid metabolism by improving intestinal morphology and altering the gut microbiota (Xie et al., 2025). Furthermore, *L.reuteri* SXDT-32 alleviates intestinal inflammation in piglets by producing short-chain fatty acids (SCFAs) that inhibit the PI3K-Akt pathway, providing an innovative approach for treating and preventing colitis caused by bacterial infections (Chen et al., 2025).

Although evidence supports the probiotic potential of *L.reuteri*, the specific impacts of early-life intervention timing and duration on the multi-omics landscape of suckling piglets remain to be fully elucidated. Therefore, this study evaluated the effects of early-life oral *L.reuteri* intervention with different durations on growth, diarrhea incidence, immunity, oxidative status, intestinal morphology, gut microbiota, ileal metabolome, and intestinal barrier markers in suckling piglets. The findings aim to provide evidence supporting the application of *L.reuteri* in early-life nutritional strategies for piglet health management.

## 2 Materials and methods

### 2.1 Bacterial preparation

The *L. reuteri* strain ATCC 53608 (CICC 6118) was obtained from the China Center of Industrial Culture Collection (CICC). The strain was cultured in de Man, Rogosa, and Sharpe (MRS) medium (Hangzhou Baisi Biotechnology Co.) at 37 °C for 20 h in anaerobic condition. Viable cell counts were determined using the agar plate count method. Before administration, the bacterial suspension was adjusted to a final concentration of 1 × 10^9^ CFU/mL with sterile MRS medium, and freshly prepared immediately before feeding to piglets to ensure the viable bacterial count.

### 2.2 Experimental animals and design

Twelve litters of piglets with similar genetic backgrounds and birth dates were selected. Litter sizes were standardized to 10 piglets per sow. Piglets were randomly assigned to three groups: Control (CK), *L.reuteri* for 3 days (D3), and *L.reuteri* for 7 days (D7), with four replicates per group (10 piglets per replicate). Piglets in the treatment groups were orally administered 2 mL of *L.reuteri* suspension (1 × 10^9^ CFU/mL) starting on day 1 after birth. The D3 group received the probiotic for three consecutive days, while the D7 group received it for seven consecutive days. The CK group received an equal volume of sterile saline. The trial lasted from birth to weaning (day 21). Piglets remained with sows and suckled freely. During the entire experimental period, all sows were fed a lactation diet, which was formulated to meet the nutritional requirements of lactating sows according to the National Research Council (N R C Council, 2012). The diet was free of probiotics, antibiotics, or any other medicinal additives, and its detailed composition and nutrient levels are shown in Table 1. Sows were individually fed twice daily at 08:00 and 17:00, with *ad libitum* access to water. All piglets received iron supplementation within the first three days after birth. No creep feed was provided to piglets during the trial; thus, they relied on sow milk as their sole source of nutrition. No antibiotics or other medicinal agents were used. Piglets were allowed to suckle freely and had *ad libitum* access to water via nipple drinkers. Standard immunization procedures were implemented throughout the entire experimental period.

**Table 1:** Diet composition and nutrient levels.

Material	Content(kg)
Corn	420
Barley	125
Biscuit meal	80
Soybean meal (46% CP)	131
Wheat flour	50
Rice bran meal	41.5
Fermented soybean meal	50
Enzymatically hydrolyzed soybean meal (CP ≥ 50%)	20
Coconut oil	12.5
Fermented mixed ingredients composed of corn (20%), soybean meal (20%) and wheat bran (60%)	30
4% Premix (Vitamins, Minerals, Ca, P, Salts and Amino Acids)	40
Total	1000

### 2.3 Sample collection

On Day 21, six piglets (*n* = 6 per group) with similar body weight, good health and no diarrhea, and a balanced sex ratio (males and females) were randomly selected from each treatment group for slaughter and sample collection. Blood samples were collected via the anterior vena cava into anticoagulant tubes. Piglets were then humanely euthanized. After slaughter, the abdominal cavity was opened, and intestinal segments (jejunum, ileum, cecum, and colon) were ligated at both ends to avoid cross-contamination. The intestinal contents were gently squeezed into sterile cryogenic tubes, immediately flash-frozen in liquid nitrogen, and then stored at −80 °C for subsequent analysis of intestinal microbiota and metabolites. Mucosa was scraped from the intestine with sterilized slides after the contents had been washed with sterile saline, and stored at −80 °C until analysis. Meanwhile, tissue samples from the duodenum, jejunum, ileum, cecum, and colon were collected, These segments were rinsed with sterile saline and fixed in 4% paraformaldehyde for histological analysis. All sampling procedures were completed within 15 minutes after slaughter to ensure sample integrity.

### 2.4 Growth Performance and Diarrhea Assessment

All piglets were weighed on the day of birth and on weaning day (day 21). The formula for average daily gain (ADG) was calculated as follows: ADG = (Weaning weight – Birth weight)/Total experimental days. To evaluate diarrhea incidence in suckling piglets, fecal consistency was observed and recorded twice daily (09:00 and 18:00) throughout the experiment using a standardized fecal scoring system (Pons et al., 2025). Diarrhea was scored based on fecal consistency as follows: 0 = normal (solid feces), 1 = soft feces, 2 = mild diarrhea (semi-liquid feces), 3 = severe diarrhea (liquid feces). Piglets with a diarrhea score ≥ 2 in two consecutive observations were classified as diarrheic. Diarrhea incidence was calculated as: Diarrhea incidence (%) = (Number of Diarrheal Piglets per Replicate)/ [Total Number of Piglets per Replicate × Trial Days] × 100.

### 2.5 Intestinal morphology

After slaughter, tissue samples were collected from the duodenum, jejunum, ileum, cecum, and mid-colon of the piglets. Three samples were randomly selected from each intestinal segment (*n* = 3) for preparing tissue sections used in intestinal morphological analysis. Fixed tissue samples were embedded in paraffin, sectioned at 5 μ*m*, and stained with hematoxylin and eosin (H and E). For each section, five non-overlapping fields were randomly selected under a light microscope at 200× magnification. Villus height and crypt depth were measured using Image *J* software. A total of 10 villi and 10 crypts were counted for each piglet.

### 2.6 Measurement of inflammatory cytokines and antioxidant indicators

Levels of cytokines (TNF-α, IL-*1*β, IL-4, IL-6, IL-10, TGF-β) and antioxidant indicators (T-AOC, T-SOD, GSH-Px, MDA) in serum and ileal mucosa were measured using ELISA kits (FANKEW, Shanghai Kexing Trading Co., Ltd., China). Ileal mucosa was homogenized in cold PBS and centrifuged to obtain supernatant. Assays were performed following manufacturer instructions, and absorbance was measured at 450 nm. Concentrations were calculated from standard curves.

### 2.7 16S rDNA sequencing and data analysis

Genomic DNA was extracted from intestinal contents using the QIA amp Fast DNA Stool Mini Kit. The full-length bacterial 16S rRNA gene was amplified using primers 27F (5′-AGRGTTYGATYMTGGCTCAG-3′) and 1492R (5′-RGYTACCTTGTTACGACTT-3′) (Souza et al., 2014). Libraries were prepared using the SMRT bell Prep Kit 3.0 (Pacific Biosciences, USA). Sequence processing and de noising were performed in QIIME2 (v2021.4) to generate amplicon sequence variants (ASVs). Alpha diversity indices (Chao1, Shannon, Simpson) were computed to assess within-sample richness and diversity. Beta diversity was assessed via principal coordinate analysis (PCoA) based on Bray-Curtis dissimilarity. Taxonomic assignment was performed using the SILVA 138 database. Differential taxa were identified using LEfSe with an LDA threshold of 4.0. Functional prediction was performed using PICRUSt2 and annotated against KEGG.

### 2.8 Metabolomic analysis

Ileal contents were analyzed for metabolites using liquid chromatography-mass spectrometry (LC-MS). Quality control (QC) samples were prepared by pooling aliquots from all samples to monitor system stability and reproducibility. Chromatographic separation and mass spectrometric detection were performed according to standard protocols. Raw LC-MS data were processed for peak detection, alignment, and normalization. Principal component analysis (PCA) and partial least squares discriminant analysis (PLS-DA) were performed to identify global metabolic differences between groups. Metabolites with variable importance in projection (VIP) > 2 were considered differential. Identified differential metabolites were further subjected to KEGG pathway enrichment analysis to determine significantly affected metabolic path.

### 2.9 RT-qPCR analysis

Total RNA was isolated from ileal mucosal samples randomly collected from 6 piglets per group (*n* = 6), with three technical replicates per sample. Extraction was performed using TRIzol reagent (Vazyme, China; Cat. No. R411). Purity was assessed by A260/ A280, and integrity was confirmed by agarose gel electrophoresis. cDNA was synthesized using a reverse transcription kit (TaKaRa, Japan; Cat. No. RR037A). RT-qPCR was performed using ChamQ SYBR qPCR Master Mix (Vazyme, China; Cat. No. Q711-03). Target genes included Claudin-3, Occludin, ZO-1, MUC1, and MUC2; β-actin served as reference gene. Primer sequences are provided in Supplementary Table 1. Relative expression was calculated using the 2^−ΔΔ^Ct method (Livak and Schmittgen, 2001), Prior to formal analysis, standard curves were generated to validate amplification efficiencies of target and reference genes. Amplification efficiencies for all genes fell within the range of 95% to 110%, meeting the criteria of the 2^−ΔΔ^Ct method. Gene expression was normalized to the reference gene, and relative expression levels were determined accordingly.

### 2.10 Western blot analysis

Ileal mucosa samples were randomly collected from three piglets in each group (*n* = 3) for protein extraction. Ileal mucosa was homogenized in RIPA buffer containing protease and phosphatase inhibitors. Protein concentration was measured using a BCA Protein Assay Kit (Beyotime, China; Cat. No. P0012). Equal amounts of protein (25 μg) were separated by SDS–PAGE and transferred onto PVDF membranes (Millipore, USA; Cat. No. IPVH09120). Membranes were blocked with 5% non-fat milk in TBST for 2 h at room temperature and then incubated overnight at 4 °C with primary antibodies against Claudin-3 (Abclonal, China; Cat. No. A24603), Occludin (Abclonal, China; Cat. No. A12621), MUC-2 (Abclonal, China; Cat. No. A4767) and β-actin (Abclonal, China; Cat. No. AC050) as the internal control. After washing, membranes were incubated with HRP-conjugated secondary antibodies for 1 h at room temperature. Protein bands were visualized using enhanced chemiluminescence (ECL) reagents and quantified by ImageJ software.

### 2.11 Statistical analysis

Data were tested for normality by the Shapiro-Wilk test and for homogeneity of variances by Levene's test. Statistical comparisons of measured indices among the three treatments were performed by Analysis of Variance followed by Student Neuman Keuls's multiple-comparison test. Spearman's rank correlation coefficients were calculated using the cortest function. Correlations among microbiota, metabolites and health outcomes were analyzed by Spearman's rank correlation test. Graphical representations were generated using Graph Pad Prism 9.5 and R software (v4.3.1). The threshold of significant difference was set at *P* < 0.05 (^*^P < 0.05, ^**^P < 0.01, ^***^P < 0.001, ^****^P < 0.0001).

## 3 Results

### 3.1 *L.reuteri* supplementation improves growth, reduces diarrhea incidence, and enhances intestinal morphology

**Figure 1:**
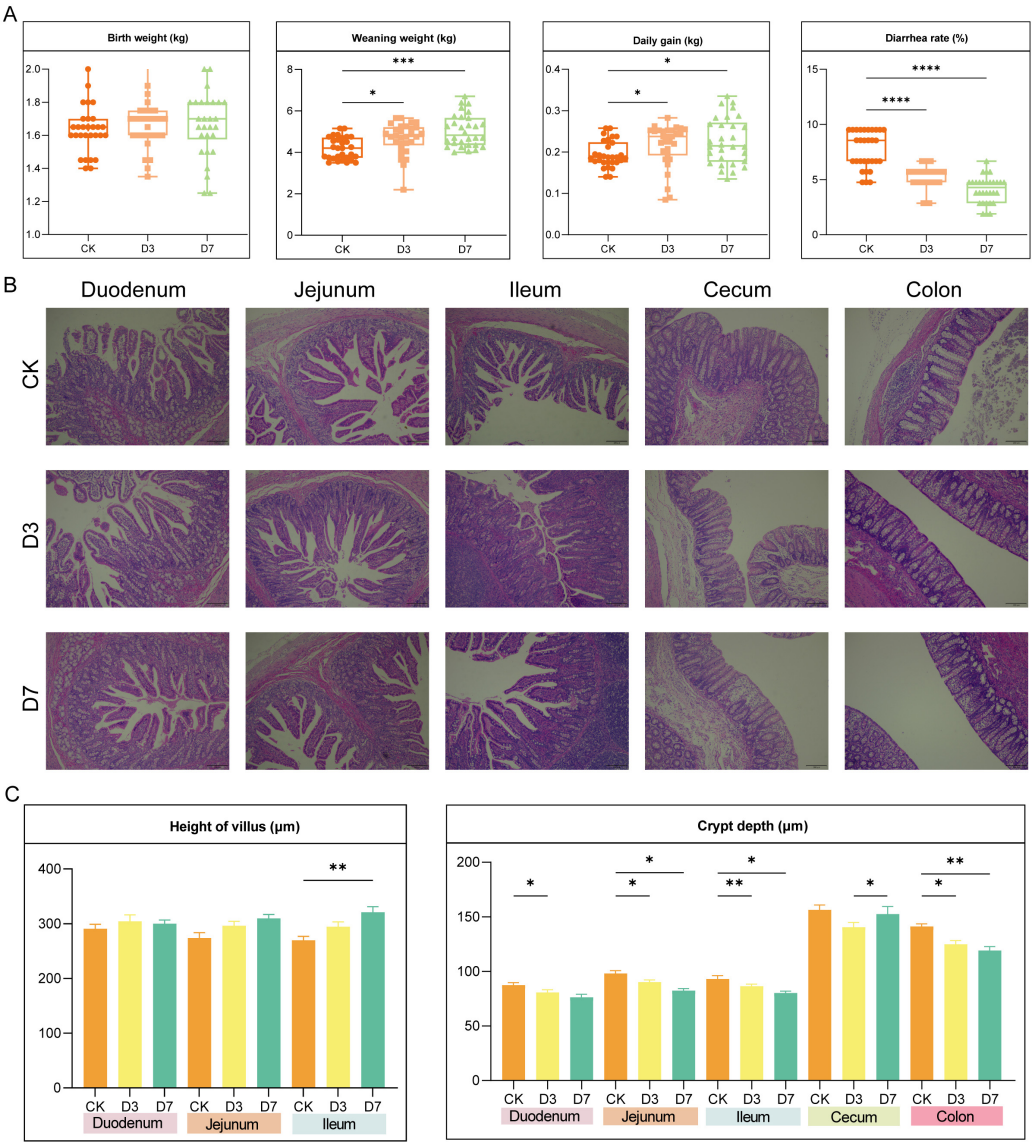
*L.reuteri* improves growth, reduces diarrhea, and enhances intestinal morphology. **(A)** Box plots of growth performance and diarrhea incidence (birth weight, weaning weight, ADG, diarrhea rate) for Control (CK), D3, and D7 groups. Significant differences between groups are indicated by asterisks (*) above the bars. **(B)** Representative H&E-stained sections of intestinal segments (Scale bar: 200 μm). **(C)** Quantification of villus height and crypt depth in duodenum, jejunum, and ileum. Data are presented as mean ± SEM. Different letters and/or asterisks indicate significant differences (*P* < 0.05).

No significant differences in birth weight were observed among groups (*P* > 0.05). However, piglets receiving *L.reuteri* (D3 and D7) exhibited significantly higher weaning weights and ADG compared to controls (*P* < 0.001). Notably, diarrhea incidence was markedly lower in the probiotic groups (*P* < 0.01) (Figure 1A). Histological analysis showed improved intestinal architecture in supplemented piglets (Figure 1B), including increased ileal villus height and reduced crypt depth in the duodenum, jejunum, ileum, and colon (Figure 1C).

### 3.2 *L.reuteri* enhances immune function and antioxidant capacity

**Figure 2:**
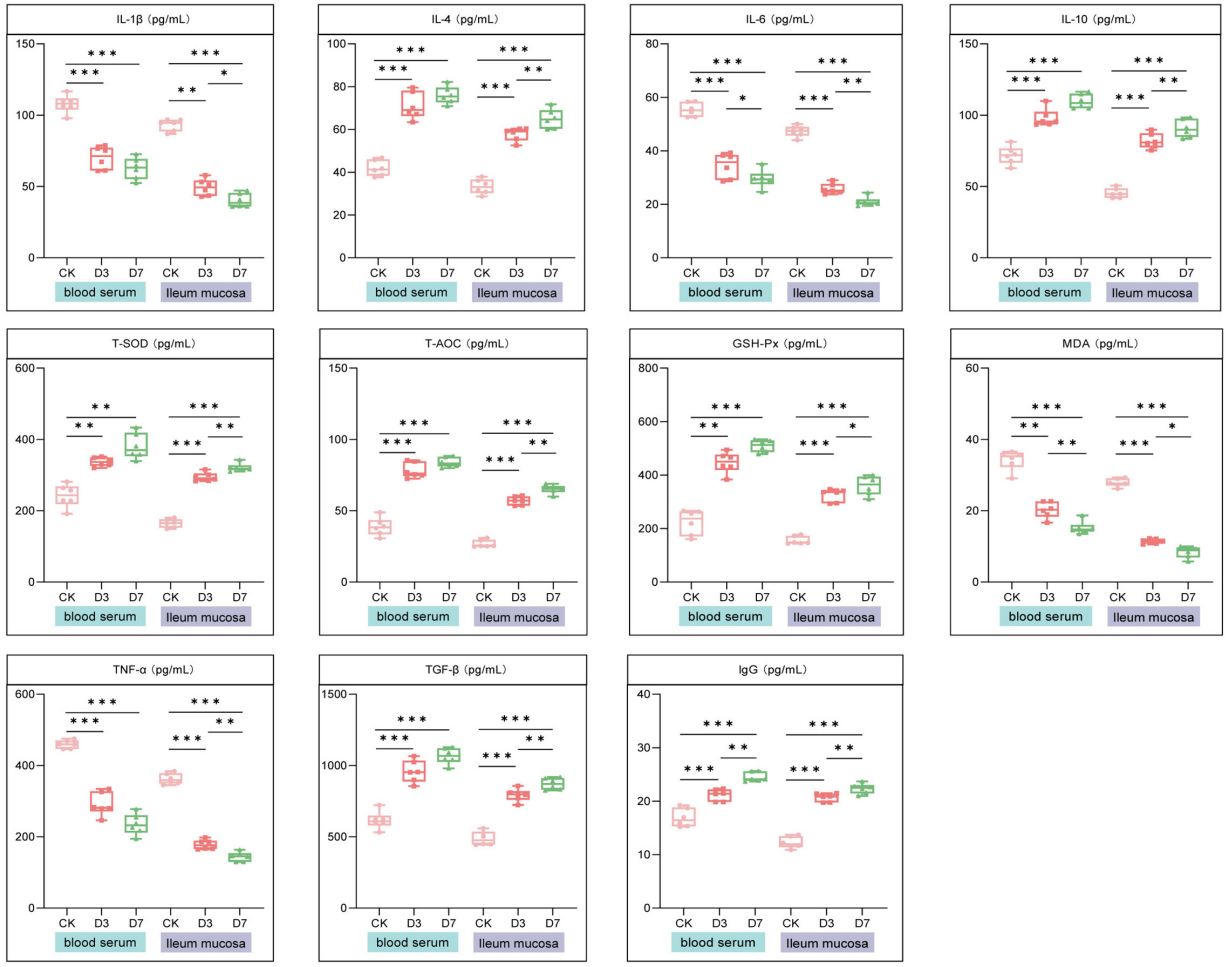
*L.reuteri* enhances immune and antioxidant status in serum and ileal mucosa. Box plots of IgG; cytokines (IL-1β, IL-6, TNF-α, IL-4, IL-10, TGF-β); antioxidant indicators (T-AOC, T-SOD, GSH-Px); and oxidative stress marker (MDA) across CON, D3, and D7 groups in serum and ileal mucosa. Asterisks indicate significant differences (*P < 0.05, **P < 0.01, ***P < 0.001).

In both serum and ileal mucosa, *L.reuteri* supplementation significantly increased levels of IgG, IL-4, and IL-10 (*P* < 0.001), while markedly reducing pro-inflammatory cytokines (IL-1β, IL-6, TNF*-*α) (*P* < 0.001). Antioxidant indicators T-SOD, T-AOC, and GSH-Px were significantly elevated, whereas MDA was significantly decreased in supplemented groups (all *P* < 0.001), indicating improved antioxidant capacity and reduced oxidative stress (Figure 2).

### 3.3 *L.reuteri* modulates gut microbiota composition

**Figure 3:**
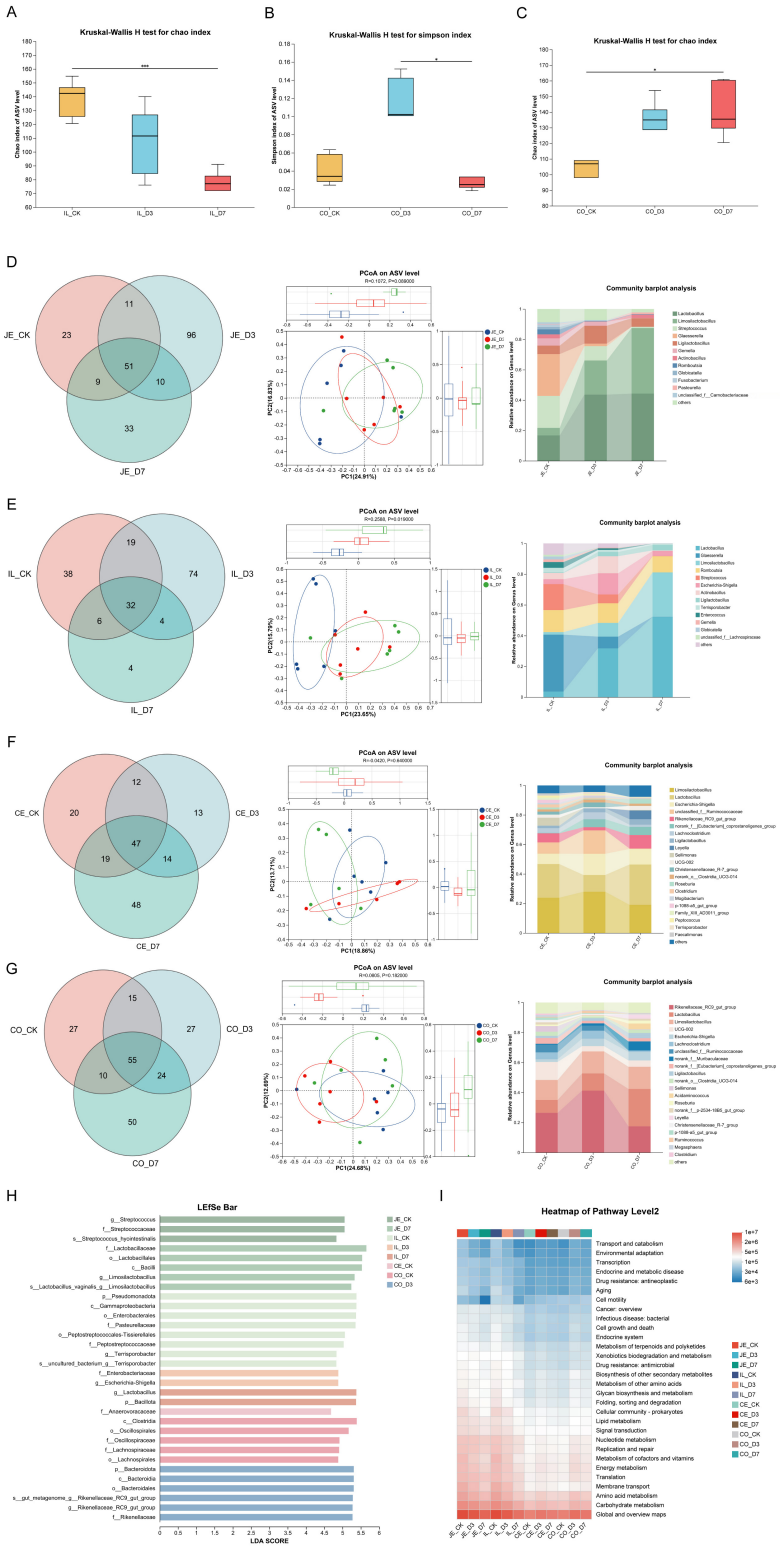
*L.reuteri* reshapes gut microbiota in different intestinal segments. **(A–C)** Alpha diversity indices (Chao1 and Simpson) across groups. **(D)** Venn diagrams showing shared and unique ASVs among groups and/or segments. **(E)** PCoA (Bray–Curtis) illustrating beta diversity, highlighting group separation in the ileum. **(F, G)** Stacked bar plots of species-level community composition across jejunum, ileum, cecum, and colon. **(H)** LEfSe results identifying discriminative taxa among groups (LDA > 4.0). **(I)** Heatmap of predicted functional pathways (PICRUSt2), annotated to KEGG/MetaCyc pathway categories.

Alpha diversity analyses indicated that *L.reuteri* supplementation significantly affected microbial richness/diversity mainly in the ileum and colon (Figures 3A–C), while other intestinal segments showed no significant differences. Beta diversity analysis demonstrated a clear separation among groups in the ileum (Figure 3E), uggesting that the ileum was the primary responsive site. Community composition plots showed that *Lactobacillus* abundance increased in the jejunum, ileum, and colon in D3 and D7 compared with CK, while cecal composition was less affected (Figures 3D–G).

LEfSe analysis showed enrichment of beneficial taxa such as Lactobacillus and *Rikenellaceae_RC9_gut_group* in supplemented piglets, whereas potentially pathogenic taxa including *Streptococcus, Pasteurellaceae*, and *Terrisporobacter* were enriched in controls (Figure 3H). Functional prediction by PICRUSt2 suggested higher predicted potential for energy metabolism, amino acid metabolism, and glycan biosynthesis/metabolism in CK jejunum and ileum compared with supplemented groups (Figure 3I).

### 3.4 *L.reuteri* alters intestinal metabolite profiles

**Figure 4:**
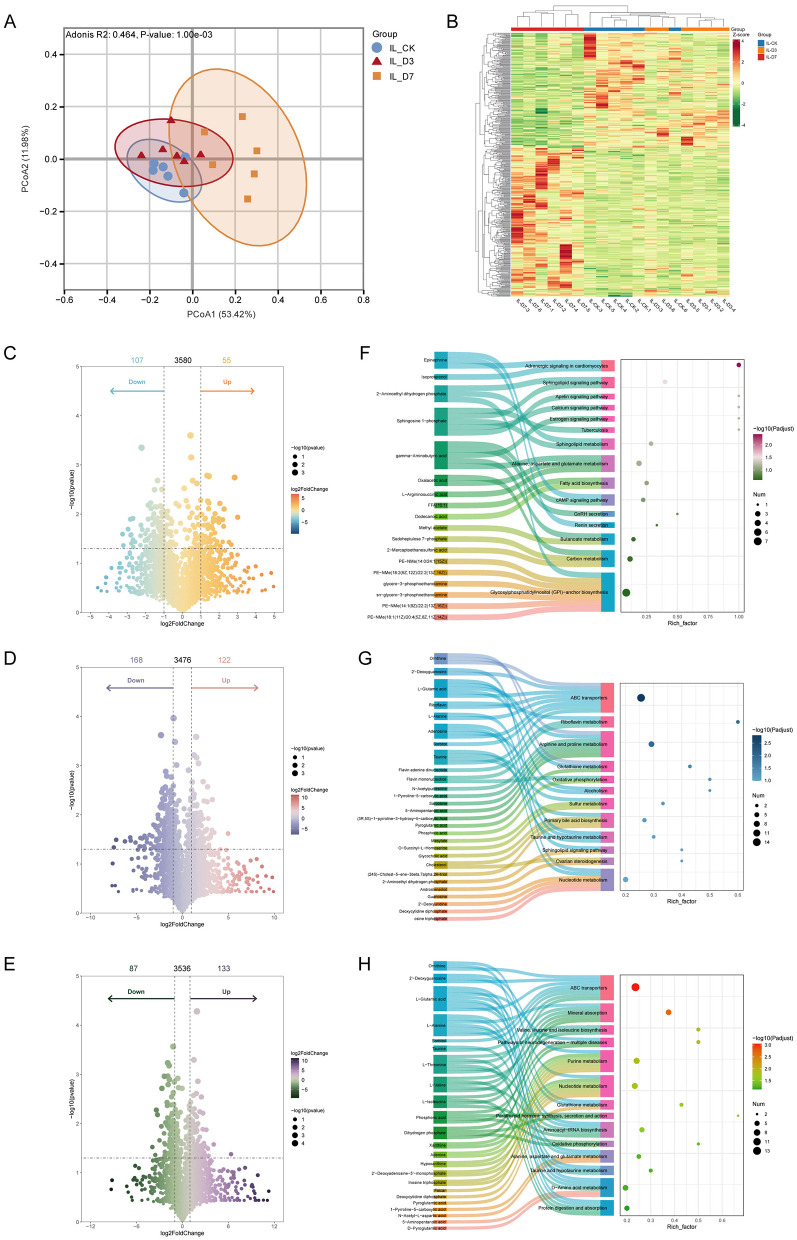
*L.reuteri* alters the ileal metabolome and enriched metabolic pathways. **(A)** PCA score plot showing separation of ileal metabolite profiles among CON, D3, and D7 groups (QC samples cluster tightly). **(B)** Heatmap of differential metabolites (hierarchical clustering) across groups. **(C–E)** Volcano plots of differential metabolites for pairwise comparisons (D3 vs CON, D7 vs CON, D7 vs D3). **(F–H)** KEGG pathway enrichment of differential metabolites for corresponding comparisons (e.g., dot plots showing significantly enriched pathways).

QC samples clustered tightly in PCA, supporting analytical stability. Ileal metabolite profiles differed substantially between supplemented and control piglets (Figure 4A), and longer supplementation (D7) produced a more pronounced metabolite shift (Figure 4B). Using PLS-DA with VIP > 2, pairwise comparisons identified 162, 290, and 220 differential metabolites. Benzoic acid, eicosadienoic acid, and indolepyruvate were significantly increased in supplemented groups, whereas peptides such as Thr-Glu-Leu-Lys, Ser-Pro-Met, and 4-hydroxyphenylacetylglycine were decreased (Figures 4C–E). KEGG enrichment analysis indicated significant effects on pathways including oxidative phosphorylation, fatty acid metabolism, nucleotide metabolism, and protein digestion and absorption (Figures 4F–H).

### 3.5 Multi-omics associations link microbiota, metabolites, and health outcomes

Correlation analyses indicated that *L.reuteri, L. amylovorus*, and *L. urinaemulieris* were negatively correlated with diarrhea incidence (*R* = −0.66, −0.75, and −0.68, respectively; *P* < 0.05), whereas *Carnobacteriaceae_bacterium_zg-C25, Helcococcus ovis*, and *Streptococcus suis* were positively correlated with diarrhea (*R* = 0.81, 0.76, and 0.70; *P* < 0.05). Metabolites Lys-Tyr (*R* = 0.79, *P* = 0.004) and Besifovir (*R* = 0.90, *P* = 0.0001) were positively correlated with diarrhea incidence, whereas benzoic acid (*R* = −0.65, *P* = 0.03) and O-arachidonoyl ethanolamine (*R* = −0.84, *P* = 0.001) were negatively correlated (Supplementary Figure 1).

Co-inertia analysis (RV = 0.420) indicated coordinated variation between microbiome and metabolome datasets. Two-omics correlation analysis suggested that Lys-Tyr, Thr-Gln-Asn, tyrosylleucine, and FAICAR were positively associated with diarrhea-associated taxa (*Carnobacteriaceae_bacterium_zg-C25, S. suis, H. ovis*) and negatively associated with *Lactobacillus* taxa. Conversely, benzoic acid and O-arachidonoyl ethanolamine showed negative associations with diarrhea-associated taxa and positive associations with *L.reuteri* and *L. amylovorus*.

**Figure 5:**
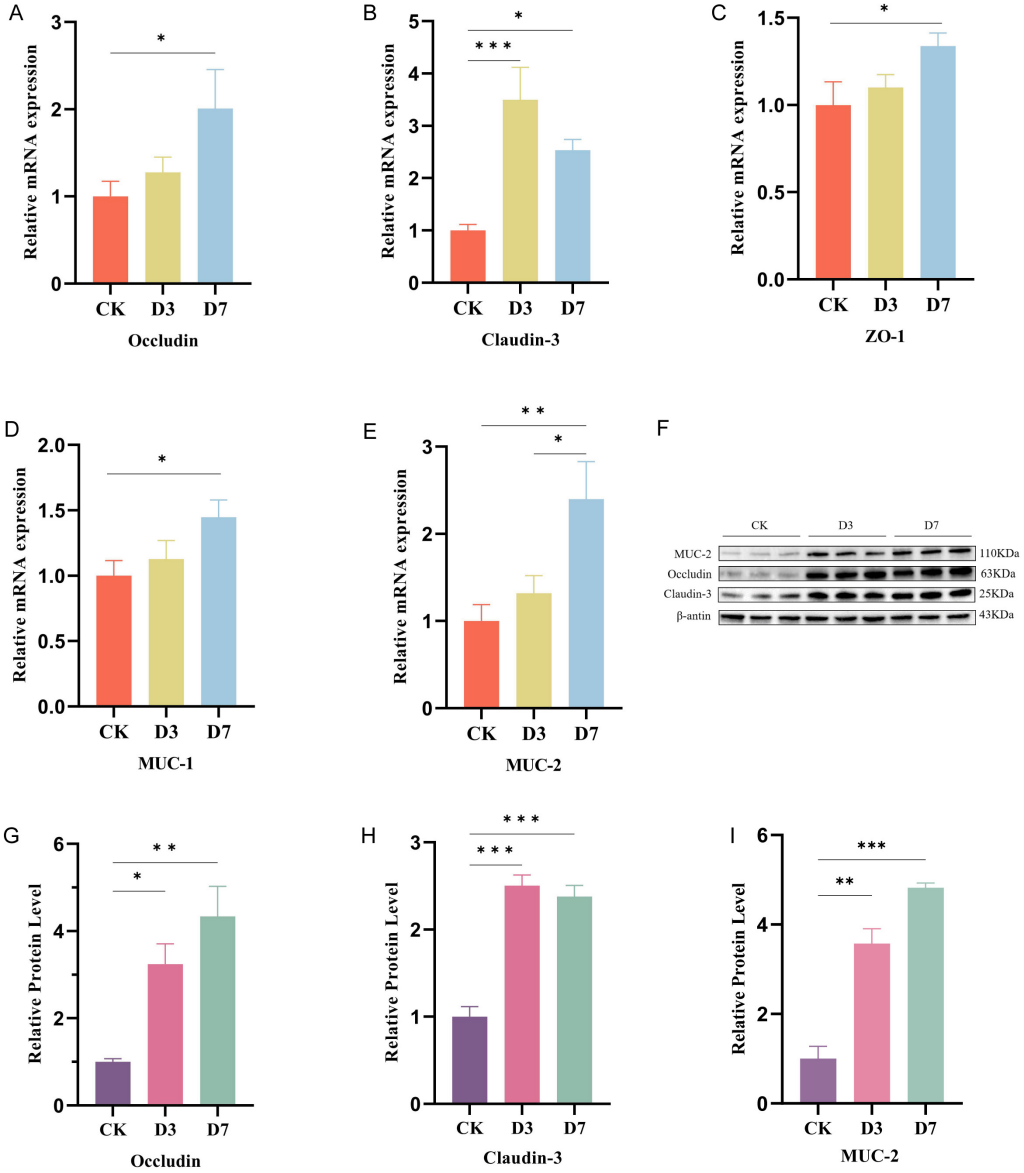
*L.reuteri* strengthens intestinal barrier gene and protein expression in ileum. **(A–E)** RT-qPCR analysis of Occludin, Claudin-3, ZO-1, MUC1, and MUC2 mRNA expression. **(F)** Representative western blots of Occludin, Claudin-3, MUC2, and β-actin. **(G–I)** Densitometric quantification of Occludin, Claudin-3, and MUC2 normalized to β-actin. Data are mean ± SEM; significance as *P < 0.05, **P < 0.01, ***P < 0.001.

Consistent with improved barrier function, mRNA expression of *Occludin, ZO-1, Claudin-3, MUC-2*, and *MUC-1* were significantly upregulated in supplemented groups (*P* < 0.05) (Figures 5A-E). Protein expression of Occludin and Claudin-3 was also significantly increased (*P* < 0.01) (Figures 5F–I).

## 4 Discussion

A growing body of evidence has demonstrated that probiotics can enhance piglet growth performance (Wang et al., 2021; Liu et al., 2022; Song et al., 2023), reduce infectious diarrhea (Wang et al., 2019a), and improve intestinal health and immune development (Wang et al., 2019a; Liu et al., 2022; Sun et al., 2022). *L.reuteri* strains exhibit probiotic properties, which can improve growth performance and alleviate diarrhea in piglets (Wang et al., 2016; Collins et al., 2023). Evidence indicates that early exposure to and colonization by an optimal microbiota contribute to reshaping the pattern of microbial succession, as well as promoting immune maturation and enhancing immune development. For example, Liu et al. (2014) found that continuous administration of *L.reuteri* I5007 for 14 days improved growth performance, reduced diarrhea incidence, and enhanced intestinal health in suckling piglets. Another study demonstrated that 20 days of consecutive intervention increased ADG and reduced diarrhea scores in suckling piglets (Liu et al., 2017). In addition, 21 days of continuous supplementation with *L.reuteri* significantly increased the final body weight and decreased the diarrhea rate of suckling piglets (Yang et al., 2020; Wang et al., 2022). These findings suggest that oral administration of *L.reuteri* can improve the growth performance of suckling piglets. Nevertheless, implementing long-term interventions from a production management standpoint proves to be exceedingly time and labor-intensive.

Accordingly, the present study aimed to determine whether shortening the intervention duration of *L.reuteri* could still improve growth performance and establish a healthy intestinal microecosystem in piglets. Our results showed that both the D3 and D7 groups significantly increased the weaning weight and ADG, and reduced the diarrhea rate of suckling piglets. The improved growth performance may be attributed to enhanced intestinal morphology, as *L.reuteri* significantly increased villus height in the ileum and decreased crypt depth in the small and large intestines, which could consequently improve nutrient digestion and absorption in piglets. This study provides a scientific basis for optimizing early intestinal microecological management in piglets.

Immunoglobulins serve as critical indicators for assessing immune status. This study demonstrated that early-life intervention with *L.reuteri* significantly elevated the levels of immunoglobulin G (IgG) in both the serum and mucosa of piglets, indicating a potential enhancement of systemic immune defense. Cytokines provide insight into the physiological and immune status of piglets from the suckling stage through weaning. Pro-inflammatory cytokines, including interferon-γ and TNF-α, play a role in the initial response and amplification of the inflammatory reaction (DiMattia et al., 2024). Probiotics have the potential to inhibit pro-inflammatory cytokines and boost the production of anti-inflammatory cytokines (Wang et al., 2019b). For example, *L.reuteri* LR1 has demonstrated the ability to reduce the gene expression of IL-1β, TNF-α, and IFN-γ, lower the protein levels of IL-6 and TNF-α, and increase TGF-β expression (Tang et al., 2021). In this study, it was observed that compared to the control group, *L.reuteri* supplementation led to elevated levels of IgG and anti-inflammatory cytokines (IL-4, IL-10, TGF-β) while decreasing pro-inflammatory cytokines (IL-1β, IL-6, TNF*-*α) in both serum and ileal mucosa. These cytokines are produced by immune cells and play a role in the host's defense against pathogens. Thus, our findings suggest that early-life intervention with *L.reuteri* can strengthen immunity and curb inflammatory reactions in suckling piglets.

The imbalance between oxidative stress and antioxidant capacity can directly affect growth performance, immune function, and metabolic homeostasis. Enzymes such as superoxide dismutase (SOD) and glutathione peroxidase (GPX) participate in the antioxidant defense system and can thus be used as biomarkers to evaluate the effects of nutritional interventions (Tariba Lovaković et al., 2021). Total antioxidant capacity (T-AOC) reflects the body's ability to respond to oxidative stress (Yang et al., 2024), whereas malondialdehyde (MDA) is a widely acknowledged biomarker of oxidative stress (Wang et al., 2025). The results of the present study demonstrated that early intervention with *L.reuteri* significantly increased the activities of T-AOC, GSH-PX, and CAT in the serum and intestinal mucosa of suckling piglets, while decreasing MDA content. These findings indicate that early-life intervention with *L.reuteri* enhances the activities of antioxidant enzymes, effectively improves the antioxidant defense system of suckling piglets, and helps reduce the diarrhea rate during the suckling periods. In agreement with the findings of the present study, various probiotics have been confirmed to improve the antioxidant capacity of piglets (Li et al., 2019; Tang et al., 2024; Ji et al., 2025).

A healthy gut microbiota is essential for body health. The gut microbiota of newborn piglets is mainly derived from the birth canal and postnatal contact with sows (Hu et al., 2023; Ruampatana et al., 2025), rendering its composition highly susceptible to external environmental factors. This susceptibility is associated with microbial instability and an increased risk of diarrhea (Dou et al., 2017). The initial gut microbiota significantly impacts the enduring health and development of piglets, where its diversity and structure are key factors influencing susceptibility to diarrhea (Ruampatana et al., 2025). Supplementation with exogenous probiotics such as Lactobacillus reuteri can increase the abundance and population of lactobacilli and other beneficial bacteria, consequently improving the gut microbiota and promoting piglet health (Al-Khazaleh et al., 2024; Zhang et al., 2025). Previous studies have reported that α-diversity, particularly the Chao index, tends to increase following probiotic supplementation (Kang et al., 2024; Liu et al., 2025), and that high microbial diversity is generally recognized as beneficial to host health and a hallmark of microbial maturation (Wang et al., 2018; Miguela-Villoldo et al., 2021; Ma et al., 2023). In this study, hindgut (cecum and colon) patterns were generally consistent with maturation-associated trends, while the ileum showed reduced richness in supplemented groups. A plausible explanation is that early colonization by *Lactobacillus* occupies ecological niches and limits overgrowth of other taxa during early life. Importantly, this segment-specific effect highlights that fecal microbiota may not fully reflect microbial changes in the small intestine, where nutrient absorption and barrier function are most active.

LEfSe results showed enrichment of beneficial *Lactobacillus* taxa and suppression of potential pathogens (e.g., *Streptococcus, Pasteurellaceae, Terrisporobacter*) in controls. The correlation analyses further supported that diarrhea incidence was negatively associated with *L.reuteri* and related *Lactobacillus* species, but positively associated with taxa such as *Streptococcus suis* and *Helcococcus ovis*. This finding is in line with previous reports showing that probiotic supplementation can effectively suppress the colonization of pathogenic bacteria in piglets (Pang et al., 2022), thereby contributing to improved intestinal health. Meanwhile, the metabolomics data indicated that peptides were higher in control piglets, which may reflect less efficient protein digestion/absorption and could provide substrates that promote dysbiosis or opportunistic pathogen proliferation in the hindgut (Bastos et al., 2020).

Metabolomics analysis identified metabolites significantly associated with the incidence of diarrhea in piglets. Among these, benzoic acid and O-arachidonoylethanolamine (OEA) showed strong negative correlations with diarrhea incidence, suggesting a potential protective role for these metabolites. As an organic acid, benzoic acid may reduce diarrhea and improve intestinal health and growth performance in piglets by lowering the intestinal pH and inhibiting bacterial proliferation (Choi and Kim, 2024). Further research has demonstrated that benzoic acid significantly reduces acetate concentrations in the duodenum and exerts potent antimicrobial effects within the gastrointestinal tract of pigs, thereby enhancing growth performance and nitrogen retention (Kluge et al., 2006; Choi and Kim, 2024). OEA belongs to a class of potent lipid mediators in mammals and is primarily involved in the regulation of lipid metabolism and energy balance through activation of peroxisome proliferator-activated receptor alpha (PPAR-α*)*. It has been shown to exert strong protective effects against inflammatory bowel disease (Grill et al., 2019). In the present study, the abundance of OEA was strongly negatively correlated with diarrhea incidence, implying that it may play an active role in alleviating intestinal inflammation and preserving epithelial integrity in piglets. The early-life intervention with *L.reuteri* may have promoted the production of such protective metabolites or slowed their depletion.

Tight junction proteins such as Occludin and Claudin-3 are key structural components of the epithelial barrier that regulate selective permeability and protect against luminal insults (Xiao et al., 2022). Mucins (MUC-1, MUC-2) further reinforce the mucosal barrier by forming protective mucus layers. These proteins maintain “selective permeability” of the epithelial barrier, providing a stable environment for nutrient absorption (Jia et al., 2021a). Functional impairment of these proteins compromises intestinal absorption, damages barrier integrity, and predisposes to disease (Zhang et al., 2019). In this study, *L.reuteri* increased both gene and protein expression of these barrier markers, supporting strengthened barrier integrity. Improved barrier function likely reduced translocation of microbial products and inflammatory activation, creating a more stable intestinal environment that supports healthy microbiota development and reduces diarrhea risk.

In conclusion, administering a shortened course of early Lactobacillus reuteri supplementation during the suckling period increases beneficial intestinal microbiota, inhibits harmful microbes, and improves intestinal barrier function and immune maturation. The strength of this study lies in demonstrating that *L.reuteri* intervention enhances disease resistance and reduces weaning stress, laying a foundation for reducing antibiotic use in the swine industry. Analysis of changes in the microbiota, metabolites, immune factors, and barrier gene expression provides insights into the effects of *L.reuteri* in neonatal piglets. Limitations include the lack of monitoring for post-weaning probiotic benefits and the potential stress associated with gavage administration. Future research should focus on validating the effects of probiotics on the growth of nursery pigs, determining optimal dosages, and developing gentler probiotic delivery methods to improve practicality in commercial swine production.

## 5 Conclusions

Early-life intervention with *L.reuteri* improves growth performance, immunity, and antioxidant capacity in suckling piglets. These benefits are mediated by a coordinated mechanism involving the reshaping of the gut microbiota, modulation of ileal metabolites, and reinforcement of intestinal barrier function. This study highlights *L.reuteri* as a potent early-life nutritional strategy to mitigate weaning-associated disorders in swine production.

## Data Availability

The data presented in the study are deposited in the MetaboLights repository, accession number MTBLS14164 (https://www.ebi.ac.uk/metabolights/MTBLS14164); the 16S rRNA gene sequencing data are deposited in the NCBI SRA repository, BioProject accession number PRJNA1405507 (https://www.ncbi.nlm.nih.gov/bioproject/PRJNA1405507).
