# Supplementary material for: Early-life intervention with Lactobacillus reuteri enhances intestinal barrier function and resilience in suckling piglets via modulation of gut microbiota and metabolites
Source: Front Microbiol. 2026 Apr 10;17:1791848. doi: 10.3389/fmicb.2026.1791848 (PMC13106610; doi:10.3389/fmicb.2026.1791848)
Supplement: Supplementary file 1 [file Supplementary_file_1.zip › Supplementary Table 1.docx]

**Supplementary Table 1. qRT-PCR primers used in this work.**

| Gene | Forward primer(5’- 3’) | Reverse primer (5’- 3’) |
| --- | --- | --- |
| *Claudin-3* | AGGACTACGTATGAGGGGGC | GACTGGTCTCGGATGCAAGG |
| *Occludin* | ACGCCACAGCCACAGCAATG | AAACGAACCCGACTAGCATCCATG |
| *ZO-1* | CAGCCCCCGTACATGGAGA | GCGCAGACGGTGTTCATAGTT |
| *MUC-1* | ACACCCATGGGCGCTATGT | GCCTGCAGAAACCTGCTCAT |
| *MUC-2* | CTGCTCCGGGTCCTGTGGGA | CCCGCTGGCTGGTGCGATAC |
| *β-actin* | ATGCTTCTAGACGGACTGCG | GTTTCAGGAGGCTGGCATGA |
