# Supplementary figures and images for: Early-life intervention with Lactobacillus reuteri enhances intestinal barrier function and resilience in suckling piglets via modulation of gut microbiota and metabolites
Source: Front Microbiol. 2026 Apr 10;17:1791848. doi: 10.3389/fmicb.2026.1791848 (PMC13106610; doi:10.3389/fmicb.2026.1791848)

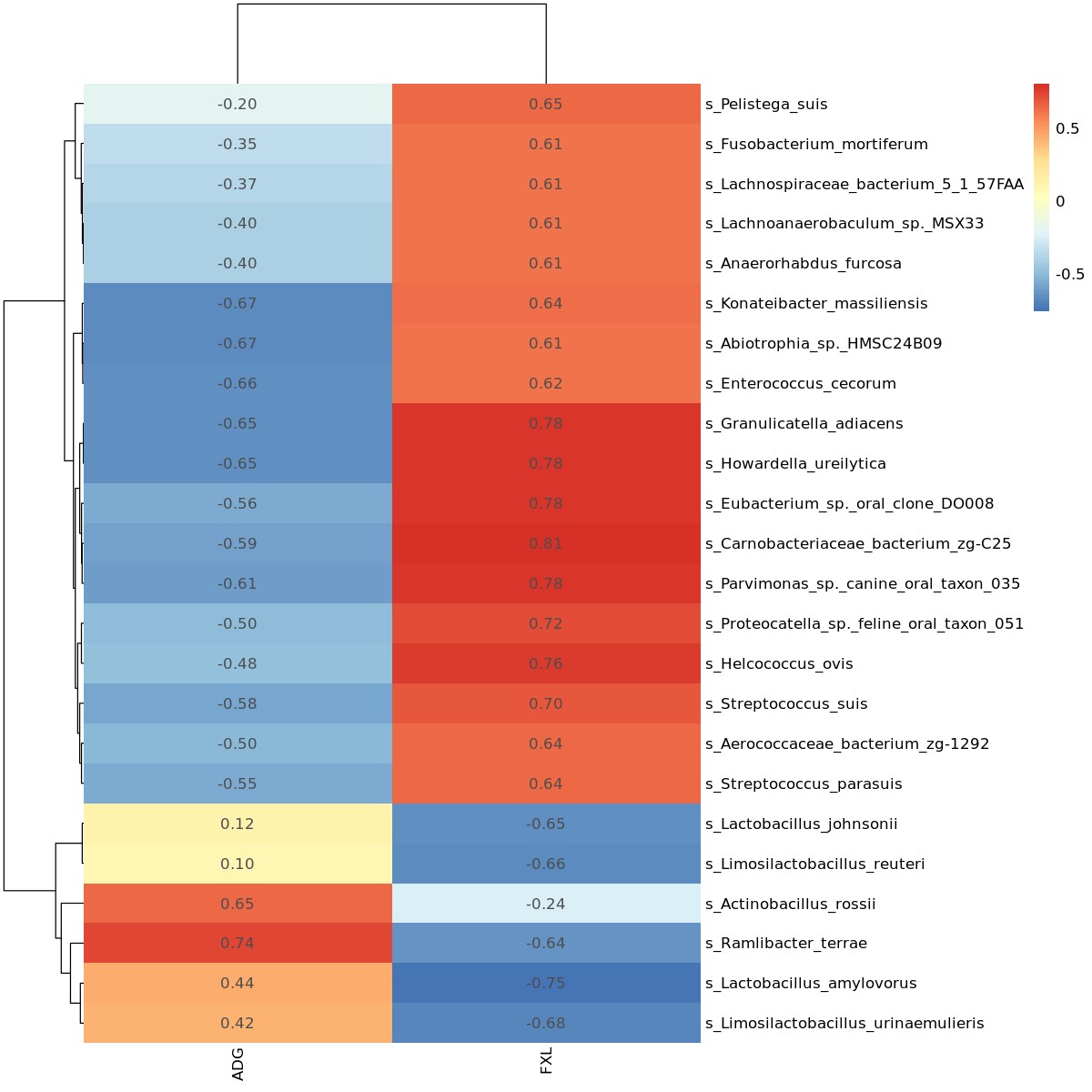

Supplement: Supplementary file 1 [file Supplementary_file_1.zip › Supplementary Figure 1 (2).jpg]

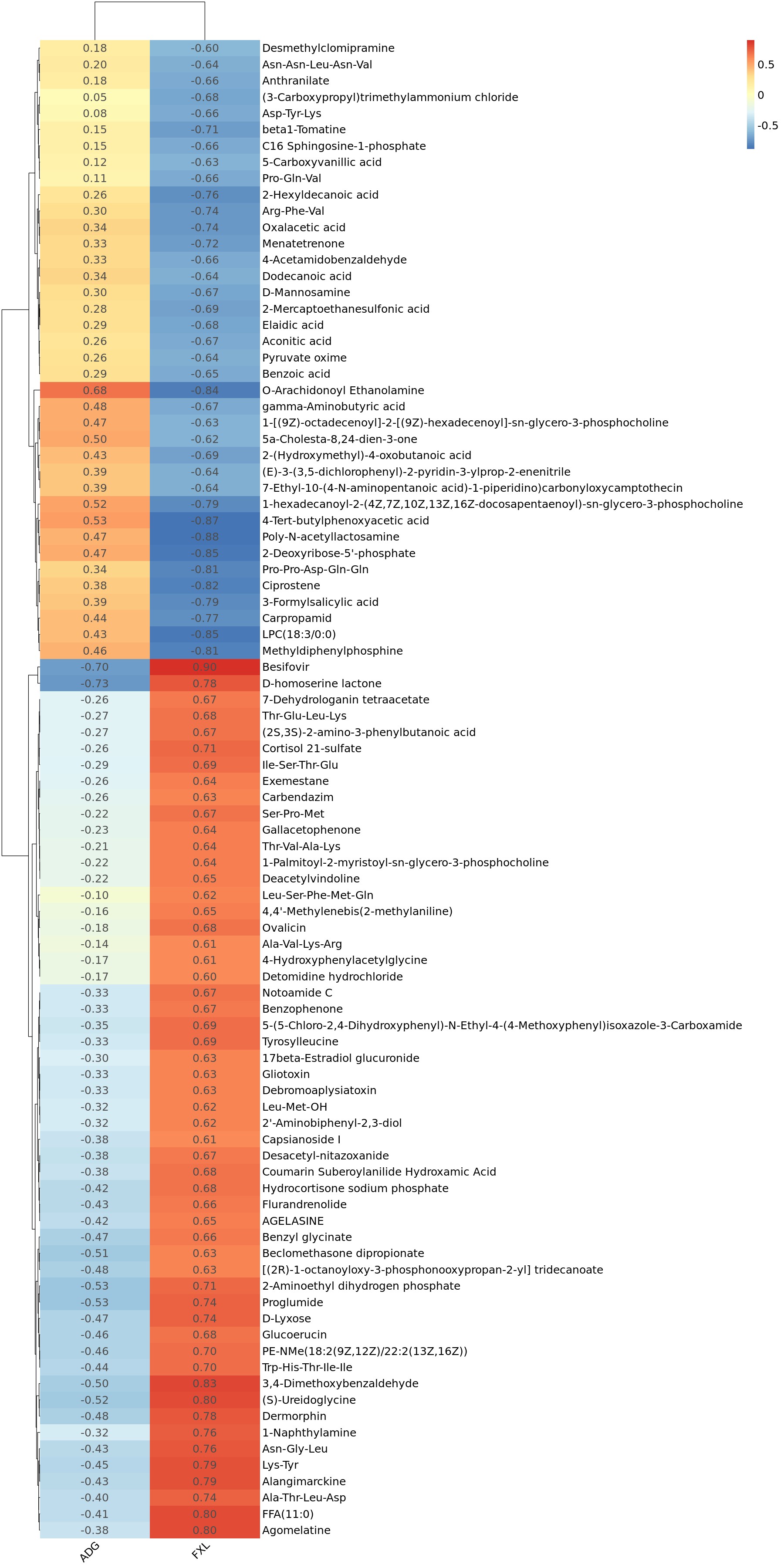

Supplement: Supplementary file 1 [file Supplementary_file_1.zip › Supplementary Figure 1 (1).jpg]
